# Correlations between histopathologic findings, serum biomarker levels, and clinical outcomes in Stevens–Johnson syndrome/toxic epidermal necrolysis (SJS/TEN)

**DOI:** 10.1038/s41598-023-40812-3

**Published:** 2023-08-21

**Authors:** Donlaporn Chuenwipasakul, Chanudda Washrawirul, Rawiphan Panpruk, Jade Wititsuwannakul, Kridipop Charoenchaipiyakul, Supranee Buranapraditkun, Vilavun Puangsricharern, Jettanong Klaewsongkram, Pawinee Rerknimitr

**Affiliations:** 1 Division of Dermatology, Department of Medicine, Chonburi Hospital, Ministry of Public Health, Chonburi, Thailand; 2 Division of Dermatology, Department of Medicine, Faculty of Medicine, Chulalongkorn University, Bangkok, Thailand; 3 Department of Ophthalmology, Faculty of Medicine, Chulalongkorn University, Bangkok, Thailand; 4 Center of Excellence for Cornea and Stem Cell Transplantation, Faculty of Medicine, Chulalongkorn University, Bangkok, Thailand; 5 King Chulalongkorn Memorial Hospital, Thai Red Cross Society, Bangkok, Thailand; 6 Division of Allergy and Clinical Immunology, Department of Medicine, Faculty of Medicine, Chulalongkorn University, Bangkok, Thailand; 7 The Skin and Allergy Research Unit, Chulalongkorn University, Bangkok, Thailand

**Keywords:** Prognostic markers, Cytokines, Immunopathogenesis

## Abstract

Stevens–Johnson syndrome (SJS) and toxic epidermal necrolysis (TEN) are severe dermatological emergencies. The role of cytokines and chemokines in the pathogenesis, progression of the disease, and histopathologic features is not fully elucidated. To address this gap, we conducted a retrospective study examining the associations between 42 serum biomarkers, histopathologic findings, and clinical outcomes in SJS/TEN patients. We reviewed the medical records of 23 patients diagnosed with SJS/TEN. Regarding histopathology, our study did not reveal any significant associations between the degree of epidermal necrosis, dermal mononuclear cell infiltration, and clinical outcomes. However, an intriguing observation was made regarding the degree of dermal infiltration of CD8 + cells, which showed a negative correlation with the severity of acute ocular complications. Notably, serum levels of IFN-γ positively correlated with the number of CD8 + cells in dermal infiltration. Additionally, higher serum levels of myeloperoxidase were associated with greater degrees of epidermal necrosis, while serum Fas ligand and stem cell factor levels were elevated in individuals with increased dermal mononuclear cell infiltration. Furthermore, the levels of S100A8/A9 were significantly correlated with the SCORTEN and mortality rate. These findings provide insights into the intricate pathogenesis of the disease.

## Introduction

Stevens–Johnson syndrome (SJS) and toxic epidermal necrolysis (TEN) are acute life-threatening mucocutaneous reactions mainly caused by medications^1^. These conditions are characterized by clinical hallmarks of confluent macules evolving into flaccid blisters and epidermal detachment associated with mucous membrane involvement. Histopathology also reveals full-thickness necrosis of the epidermis associated with mononuclear cell infiltration. SJS and TEN are regarded as different degrees of severity within the same disease spectrum, primarily differing in the extent of skin detachment. SJS is defined as the involvement of less than 10% of the body surface area (BSA), while TEN involves more than 30%. When the percentage of BSA affected is greater than 10% but less than 30%, the condition is referred to as the SJS/TEN overlap syndrome^2,3^. Although these conditions are rare, they cause significant morbidity and mortality as well as impose a major economic burden on the health care system^4,5^. Therefore, early diagnosis and risk stratification constitute a crucial part of patient evaluation and management^6^.

Currently, diagnosis of SJS/TEN is typically based on the morphology and extent of lesions in the context of previous drug exposure or illness^7^. Histological examination can aid in the diagnosis by identifying characteristic features such as epidermal necrosis with slight to absent cellular infiltration and separation of the epidermis at the dermo-epidermal junction^8^ However, early identification of SJS/TEN remains challenging because the initial clinical signs of severe drug eruptions resemble those of non-severe ones^9^. Additionally, increasing evidence suggests that the traditional scoring system used to assess SJS/TEN severity, known as the Severity-of-Illness Score for toxic epidermal necrolysis (SCORTEN), overestimates mortality in the contemporary population^10^. The association between histopathology and clinical severity is still controversial, and there is a lack of extensive immunohistochemistry studies in SJS/TEN.

In a previous study, we identified that S100A8/A9 and granulysin levels may help predict the occurrence of severe ocular complications in SJS/TEN^11^. However, the role of cytokines and chemokines in association with histopathologic features and the progression of the skin disease is not fully understood. Hence, the objective of the present study is to investigate the correlations among cutaneous histopathologic findings, including an extensive immunohistochemistry study, serum biomarker levels, ocular involvement, and clinical outcomes in SJS/TEN patients. A better understanding of disease pathophysiology may aid in the discovery of novel targeted therapies.

## Results

### Patient characteristics

A total of 23 SJS/TEN patients with available skin biopsy specimens were included in the study. The diagnosis of SJS/TEN in each case was confirmed by the Registry of Severe Cutaneous Adverse Reaction (RegiSCAR) criteria. All patients were part of the Thai Severe Cutaneous Adverse Reactions (ThaiSCAR) cohort registered at ClinicalTrials.gov (NCT02574988). Among these patients, 11 (47.8%) were male, and 12 (52.2%) were female. The mean age was 49.3 years (SD ± 15.7). The mean ± SD time from the disease onset to skin biopsy was 4.82 ± 2.40 days. The SCORTEN score was 0–1 in 8 cases (34.8%), 2–3 in 12 cases (52.2%), and 4–5 in 3 cases (13.0%). Ocular involvement was observed in 21 out of 23 patients (91.3%). The acute ocular complications were classified, as previously described, into four grades: noninvolvement (0), hyperemia (1), either epithelial defect or pseudomembrane formation (2) and both epithelial defect and pseudomembrane formation (3)^12^. The acute ocular severity score was grade 0 in 2 cases (8.7%), grade 1 in 9 cases (39.1%), grade 2 in 4 cases (17.4%), and grade 3 in 8 cases (34.8%). Most patients had a body surface area (BSA) of skin detachment less than 10% (19/23, 82.6%). The clinical findings and histology are summarized in Table 1. Among all the culprit drugs in this study, antibacterial agents including trimethoprim-sulfamethoxazole (26.1%) were the most common cause, followed by antiepileptic agents (21.8%) and allopurinol (21.8%). There were 21 patients (91.3%) with underlying diseases, including human immunodeficiency virus (HIV) infection (6 cases; 26.1%), connective tissue disease (3 cases; 13%), and malignancy (4 cases; 17.4%). Hospital mortality was 26.1% (6/23) among the study population.

**Table 1:** Demographic, clinical characteristics, and histologic findings of participants (n = 23).

Characteristics	N (%)
Sex	
Male	11 (47.8%)
Female	12 (52.2%)
Age, mean	49.3 year (SD ± 15.7)
Underlying disease	
Essential hypertension	8 (34.8%)
Diabetes mellitus type 2	6 (26.1%)
HIV infection	6 (26.1%)
Pulmonary tuberculosis	6 (26.1%)
Dyslipidemia	5 (21.7%)
Malignancy	4 (17.4%)
Cardiac disease	3 (13%)
Chronic kidney disease	3 (13%)
Connective tissue disease	3 (13%)
Psychiatric disorders	3 (13%)
Benign brain tumor	2 (8.7%)
Cerebrovascular disease	2 (8.7%)
Chronic hepatitis B virus infection	2 (8.7%)
Epilepsy	2 (8.7%)
Gouty arthritis	2 (8.7%)
Hepatitis C cirrhosis	2 (8.7%)
Mitochondrial disease	1 (4.3%)
BSA%	
< 10%	19 (82.6%)
10–30%	4 (17.4%)
> 30%	0
SCORTEN	
0	3 (13.0%)
1	5 (21.7%)
2	7 (30.4%)
3	5 (21.7%)
4	2 (8.7%)
5	1 (4.4%)
Eye severity	
0	2 (8.7%)
1	9 (39.1%)
2	4 (17.4%)
3	8 (34.8%)
Treatment*	
Supportive	7 (30.4%)
Systemic corticosteroid	14 (60.9%)
Cyclosporine	2 (8.7%)
IVIG	1 (4.3%)
Death	
Yes	6 (26.1%)
No	17 (73.9%)
Cause of death	
Sepsis	4 (66.7%)
Acute respiratory distress syndrome	1 (16.7%)
Malignancy	1 (16.7%)
Epidermal necrosis	
Mild	8 (34.8%)
Moderate	6 (26.1%)
Extensive	9 (39.1%)
Upper dermal mononuclear cell infiltration	
Mild	3 (13.0%)
Moderate	16 (69.6%)
Extensive	4 (17.4%)

*52.17% of the patients underwent skin biopsy, and 38.10% had serum taken for biomarker analysis while they were on systemic treatment.

### Histologic and immunohistochemistry findings

The histologic data collections included epidermal necrosis, dermal inflammatory cell infiltrations, and peri-appendageal inflammatory cell infiltrations. The extents of inflammatory cell infiltrations in each area and the degree of epidermal necrosis were graded into mild, moderate, and extensive. All 23 slides with hematoxylin and eosin (H&E) stain were critically reviewed and graded by three certified pathologists.

The histologic and immunohistochemistry findings revealed that the degree of epidermal necrosis was mild in 8 cases (34.8%), moderate in 6 cases (26.1%), and extensive in 9 cases (39.1%). The extent of dermal mononuclear cell infiltration was mild in 3 cases (13.0%), moderate in 16 cases (69.6%), and extensive in 4 cases (17.4%). The association between histopathologic features and clinical data is reported in Tables 2 and 3. Both the degree of epidermal necrosis and the number of upper dermal mononuclear cell infiltration were not significantly associated with SCORTEN, severity of ocular involvement, or the extent of body surface area involved. The in-hospital mortality rate was higher among patients with extensive epidermal necrosis (3/9, 33.3%) compared to patients with mild or moderate epidermal necrosis (3/14, 21.4%) but the difference was not statistically significant. Other inflammatory cells, such as neutrophils and eosinophils, in the upper dermis and adnexal necrosis were only sporadically identified in tissue samples. The presence of a smaller number of peri-appendageal inflammatory cell infiltration was also observed in the lower dermis.

**Table 2:** The correlations between severity of epidermal necrosis, upper dermal mononuclear cell infiltration, and clinical outcomes in SJS/TEN.

	Epidermal necrosis severity				Upper dermal mononuclear infiltration severity			
	Mild (n = 8)	Moderate (n = 6)	Extensive (n = 9)	***P***-value	Mild (n = 3)	Moderate (n = 16)	Extensive (n = 4)	*P*-value
SCORTEN, n (%)								
0–1	3 (37.5)	2 (33.3)	3 (33.3)	1.000	2 (66.7)	6 (37.5)	0 (0.0)	0.455
2–3	4 (50.0)	3 (50.0)	5 (55.6)		1 (33.3)	8 (50.0)	3 (75.0)	
4–5	1 (12.5)	1 (16.7)	1 (11.1)		0 (0.0)	2 (12.5)	1 (25.0)	
Eye severity, n (%)								
0–1	4 (50.0)	3 (50.0)	4 (44.4)	1.000	0 (0.0)	9 (56.2)	2 (50.0)	0.341
2–3	4 (50.0)	3 (50.0)	5 (55.6)		3 (100.0)	7 (43.8)	2 (50.0)	
Death, n (%)	2 (25.0)	1 (16.7)	3 (33.3)	0.850	1 (33.3)	5 (31.3)	0 (0.0)	0.611

*Statistically significant at a 0.05 level (α = 0.05).

**Table 3:** The correlations between immunohistochemistry staining and clinical outcomes in SJS/TEN.

	CD4 IHC cutoff point: 21 positive cells/mm^2^		*P*-value	CD8 IHC cutoff point: 19 Positive Cells/mm^2^		*P*-value	CD56 IHC cutoff point: 16 positive cells/mm^2^		*P*-value
	Low (n = 12)	High (n = 11)		Low (n = 12)	High (n = 11)		Low (n = 12)	High (n = 11)	
SCORTEN, n (%)									
0–1	6 (50.0)	2 (18.2)	0.360	6 (50.0)	2 (18.2)	0.360	4 (33.3)	4 (36.4)	0.856
2–3	5 (41.7)	7 (63.6)		5 (41.7)	7 (63.6)		7 (58.3)	5 (45.5)	
4–5	1 (8.3)	2 (18.2)		1 (8.3)	2 (18.2)		1 (8.3)	2 (18.2)	
Eye severity, n (%)									
0–1	4 (33.3)	7 (63.6)	0.220	3 (25.0)	8 (72.7)	0.039*	7 (58.3)	4 (36.4)	0.414
2–3	8 (66.7)	4 (36.4)		9 (75.0)	3 (27.3)		5 (41.7)	7 (63.6)	
Death, n (%)	2 (16.7)	4 (36.4)	0.371	3 (25.0)	3 (27.3)	1.000	3 (25.0)	3 (27.3)	1.000

	CD68 IHC cutoff point: 21 positive cells/mm^2^		*P*-value	CD117 IHC cutoff point: 6 positive cells/mm^2^		*P*-value	GB IHC cutoff point: 1 positive cells/mm^2^		*P*-value
	Low (n = 12)	High (n = 11)		Low (n = 13)	High (n = 10)		Low (n = 16)	High (n = 7)	
SCORTEN, n (%)									
0–1	5 (41.7)	3 (27.3)	0.733	5 (38.5)	3 (30.0)	0.719	5 (31.3)	3 (42.9)	0.830
2–3	6 (50.0)	6 (54.5)		7 (53.8)	5 (50.0)		9 (56.3)	3 (42.9)	
4–5	1 (8.3)	2 (18.2)		1 (7.7)	2 (20.0)		2 (12.5)	1 (14.2)	
Eye severity, n (%)									
0–1	6 (50.0)	5 (45.5)	1.000	3 (23.1)	8 (80.0)	0.012	8 (50.0)	3 (42.9)	1.000
2–3	6 (50.0)	6 (54.5)		10 (76.9)	2 (20.0)		8 (50.0)	4 (57.1)	
Death, n (%)	3 (25.0)	3 (27.3)	1.000	4 (30.8)	2 (20.0)	0.660	6 (37.5)	0 (0.0)	0.124

Data were analyzed with Chi-square test (Exact method).

* Statistically significant at a 0.05 level (α = 0.05).

**Figure1:**
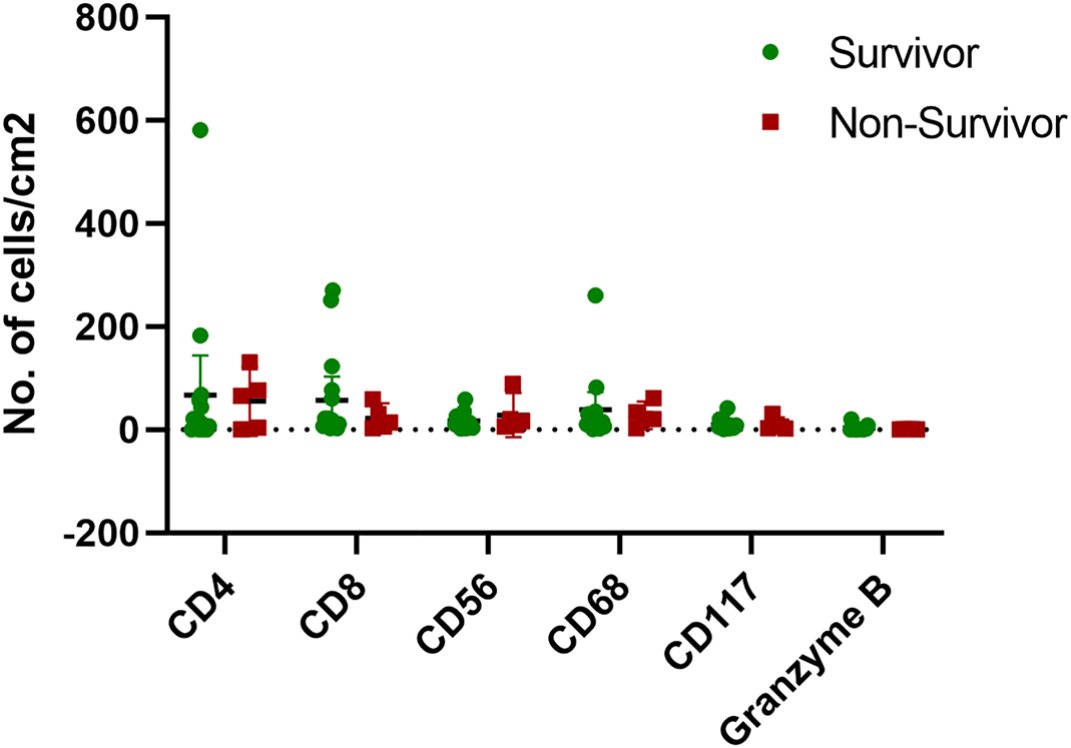
Number of inflammatory cells in the dermis, the immunohistochemical study.

An immunohistochemical analysis was performed to determine the CD4, CD8, CD56, CD68, CD117 and granzyme B-expressing cells in tissue biopsies. Then, Aperio Scanscope CS system was used to scan the slides and the whole areas of the dermis were selected to quantify the positivity of the staining. Median values of CD4, CD8, and CD68-expressing cells were higher compared to CD56, CD117 and granzyme B. The comparisons of the cell densities between the disease survivors and non-survivors did not demonstrate a significant difference and are shown in Fig. 1.The severity of dermal infiltration of CD8-expressing cells showed a significant inverse association with ocular severity (*p* = 0.039): 75.0% (9/12) of patients with a low level of CD8-expressing cells had severe to very severe ocular involvement (grade 2 or 3), while 27.3% (3/11) of patients with a high level of infiltration had severe to very severe ocular involvement (grade 2 or 3).

### The correlations between serum biomarker levels and histopathological findings

**Figure 2:**
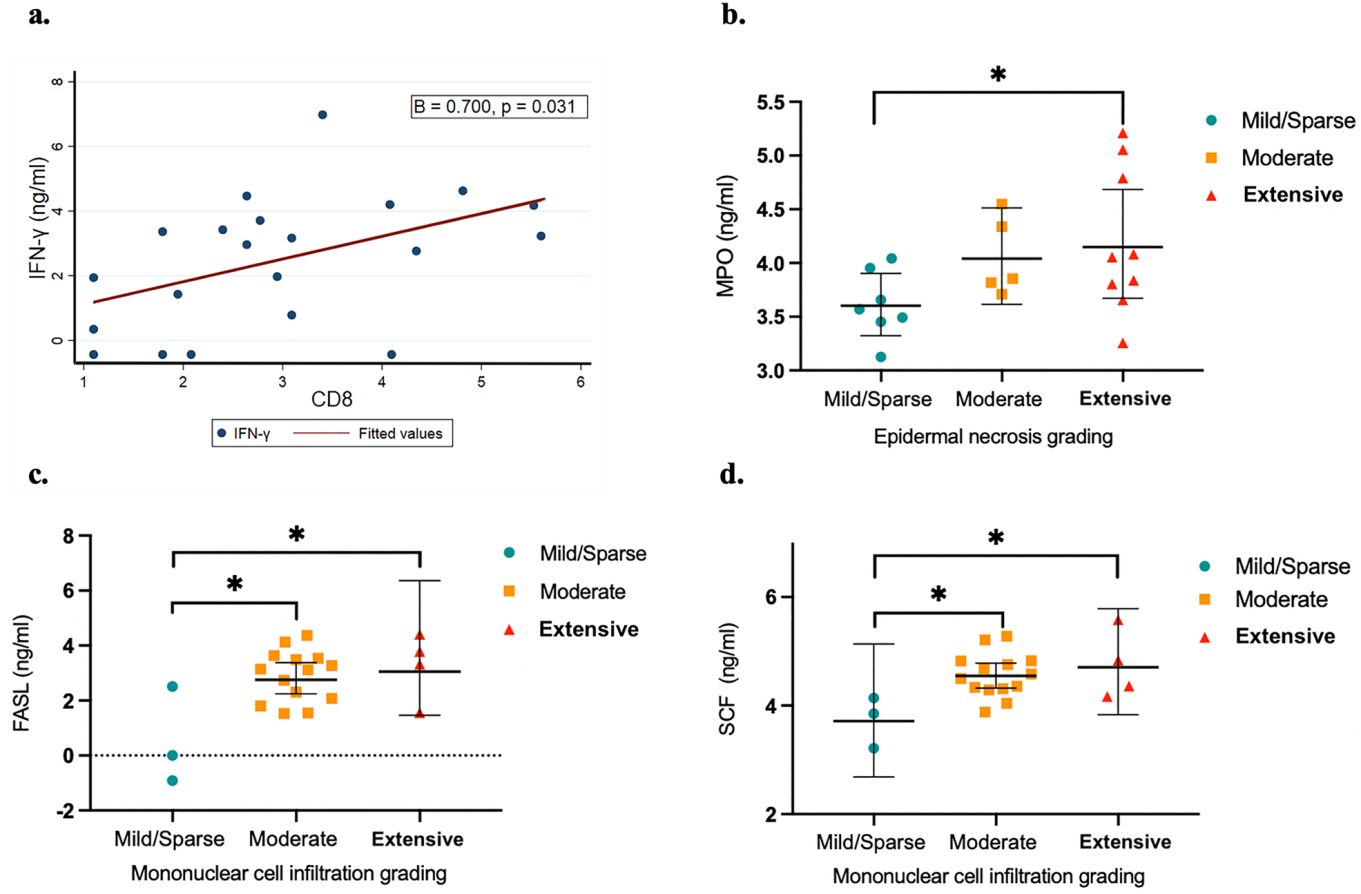
The correlations between serum biomarker levels and histopathological outcomes in SJS/TEN. (**a**) Linear regression analysis using log-transformed values between the number of cells positive for CD8 (cell/mm^2^) and serum IFN-γ levels. (**b**) Scatter plot graphs of serum MPO levels (geometric means with 95% confidence intervals) according to each grade of epidermal necrosis. (**c**) Scatter plot graphs of serum sFASL levels (geometric means with 95% confidence intervals) according to each grade of mononuclear cell infiltration in the dermis. (**d**) Scatter plot graphs of serum SCF levels (geometric means with 95% confidence intervals) according to each grade of mononuclear cell infiltration in the dermis. *p < 0.05.

The serum samples of the patients were collected within a maximum of 3 days following admission. However, the mean ± SD time from the disease onset to serum collection was 4.35 ± 2.46 days. The serum was analyzed using a bead-based multiplex immunoassay. Out of 23 patients, 21 had complete biomarker data and were included in the analysis. From a total of 42 serum biomarkers, we found that interferon-gamma (IFN-γ), myeloperoxidase (MPO), soluble Fas ligand (sFasL), and stem cell factor (SCF) were highly related to the histologic features of SJS/TEN. Serum IFN-γ levels were positively correlated with number of CD8 + cells (cell/mm^2^) in the dermal infiltration (β = 0.700, *p* = 0.031) (Fig. 2a). Serum MPO levels were also significantly associated with the degree of epidermal necrosis (Fig. 2b). The geometric mean (GM) of MPO serum concentration was 1.79 times higher [geometric mean ratio (GMR), 1.79; 95% CI 1.04–3.08; *p* = 0.037] in the group with extensive epidermal necrosis (GM, 66.3; %CV, 75.5) than in the group with mild epidermal necrosis (GM, 37.1; %CV, 31.9). Moreover, serum sFasL and SCF levels were found to be higher in those with more mononuclear cell infiltration in the dermis. When comparing groups with different degrees of dermal mononuclear cell infiltration, those with moderate infiltration (GM, 18.3; %CV, 116.8) had 10.85 times higher geometric mean serum concentration of sFasL (GMR, 10.85; 95% CI 2.46–47.10; *p* = 0.003) compared to those with mild infiltration (GM, 1.7; %CV, 472.4). In those with extensive infiltration (GM, 26.3; %CV, 186.6), the concentration increased to 15.56 times (GMR, 15.56; 95% CI 2.63–91.15; *p* = 0.010)** (**Fig. 2c). In accordance with the SCF, the serum concentration of those with moderate infiltration (GM, 95.8; %CV, 41.8) was 2.29 times higher (GMR, 2.29; 95% CI 1.24–4.20; *p* = 0.011) than those with mild infiltration (GM, 41.9; %CV, 50.2) and increased to 2.72 times (GMR, 2.72; 95% CI 1.31–5.64; *p* = 0.010) for those with extensive infiltration (GM, 114.1; %CV, 69.6) (Fig. 2d).

### The correlations between serum biomarker levels and clinical outcomes

**Figure 3:**
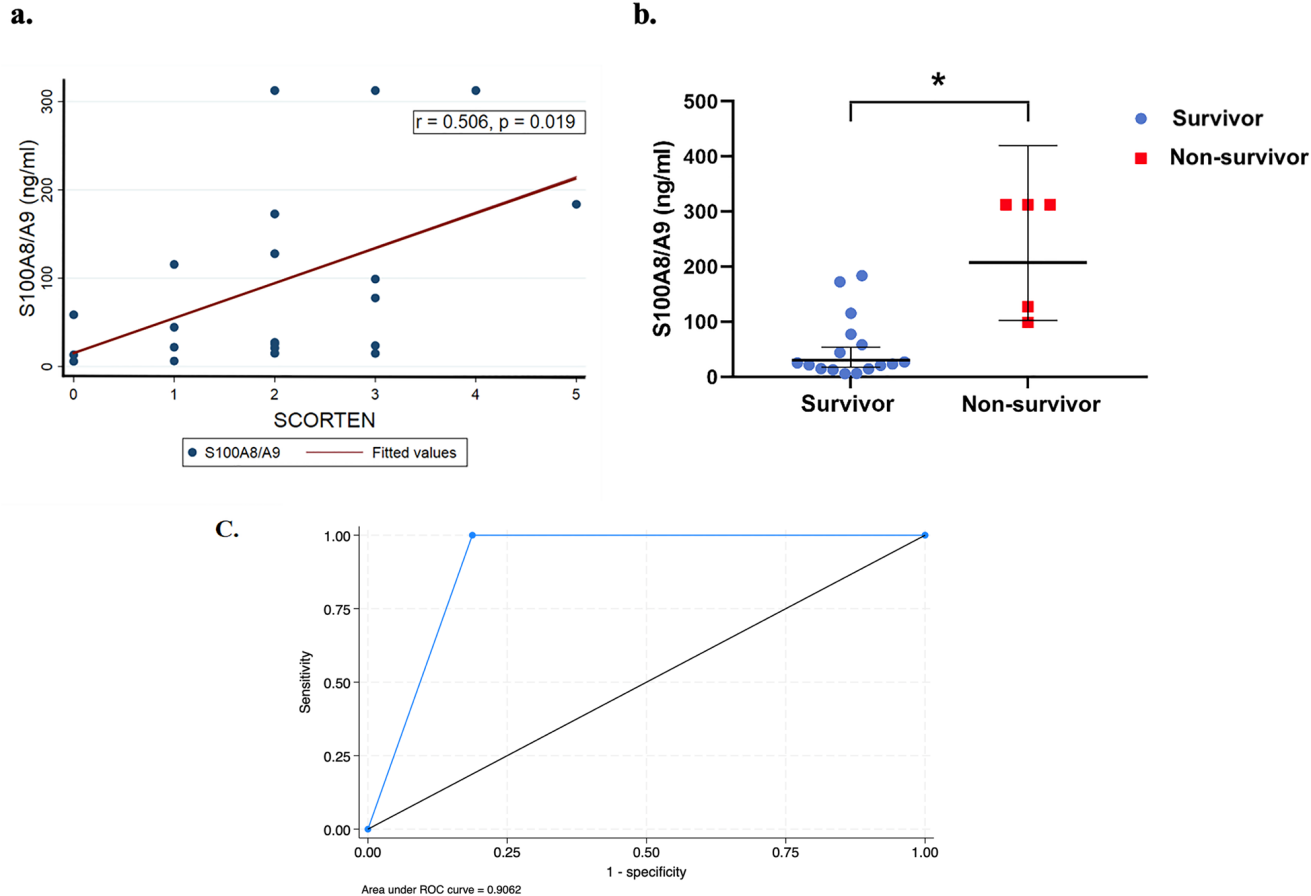
The correlations between serum biomarker levels and clinical outcomes in SJS/TEN. (**a**) Spearman correlation between serum S100A8/A9 levels and the SCORTEN severity score. (**b**) Scatter plot graphs of serum S100A8/A9 levels (geometric means with the 95% confidence interval) between SJS/TEN survivors and non-survivors. *p < 0.05 (**c**) ROC curve analysis of serum S100A8/A9 levels to identify an optimal cut-off point to predict mortality. Cut off value is 88.375. sensitivity = 100% (95% CI 47.8, 100.0) Specificity = 81.3% (95% CI 54.4, 96.0) Positive predictive value = 62.5% (95% CI 24.5, 91.5%) AUC ROC 0.9062 (95% CI 0.81, 1.0).

Serum levels of S100A8/A9 were positively correlated with the SCORTEN severity score (Spearman’s rho 0.506; *p* = 0.019) (Fig. 3a). Additionally, elevated levels of S100A8/A9 were associated with in-hospital mortality of SJS/TEN. The geometric mean serum concentration of S100A8/A9 in the non-survivor group (GM, 207.7; %CV, 61.5) was 6.8 times higher (GMR, 6.80; 95% CI 2.38–19.16; *P* = 0.001) than in the survivor group (GM, 30.7; %CV, 142.8) (Fig. 3b). ROC curve analysis with the Liu method was used to identify an optimal cut-off point of S100A8/A9 to predict mortality. When the cut-off value of 88.38 was used, the sensitivity was 100% (95% CI 47.8, 100.0), specificity was 81.3% (95% CI 54.4, 96.0), the positive predictive value was 62.5% (95% CI 24.5, 91.5%), and the AUC ROC was 0.9062 (95% CI 0.81, 1.0) (Fig. 3c). The mean ± SD time from disease onset to serum sampling for those who survived versus those who died was 4.63 ± 2.55 days versus 3.6 ± 1.81 days, respectively. Additionally, the mean time from obtaining the serum samples to death was 49.6 ± 35.80 days.

The comparison of GMs of all serum biomarker levels between the survivors and non-survivors was shown in Table 4. Apart from S100A8/A9, the GMs of granulocyte-colony stimulating factor (G-CSF) and erythropoietin (EPO) in the non-survivor group were significantly higher than in those who survived (GMR, 4.80; 95% CI 1.37–16.91; *p* = 0.017 and GMR, 2.0; 95% CI 1.07–3.64; *p* = 0.03, respectively).

**Table 4:** The comparison of geometric means (GM) of serum biomarkers levels between SJS/TEN survivors and non-survivors.

Biomarkers	GM, ng/ml (%CV)		GMR	95% CI	*p*-value
	Survivors	Non-survivors			
TGF-b1	0.9 (157.9)	2.6 (2024.2)	2.9	0.58–14.55	0.181
IL-18	95.2 (462.1)	526.2 (1880.5)	5.5	0.70–43.33	0.099
IP-10	3047.9 (189.9)	7735.8 (46.5)	2.5	0.77–8.41	0.12
MCP-1	160.3 (101.6)	355.1 (30.6)	2.2	0.98–5.01	0.055
sFASL	16.9 (226.8)	7.6 (261.2)	0.4	0.10–1.94	0.266
IL-15′	1.7 (728.0)	8.3 (4798.6)	4.9	0.47–50.99	0.173
Rantes	551.3 (1192.6)	68.8 (314,596.8)	0.1	0.01–2.27	0.149
IL-23	0.5 (51.3)	1.0 (184.5)	1.8	0.85–3.87	0.114
Granulysin	691.1 (93.9)	1498.6 (205.9)	2.2	0.81–5.82	0.117
IL-6	36.3 (631.6)	165.8 (93.3)	4.6	0.70–29.86	0.106
IL-10	2.7 (273.3)	10.4 (114.3)	3.8	0.89–16.55	0.07
IFN-γ	9.7 (382.5)	22.5 11,815.8	2.3	0.26–20.88	0.429
TNF-α	1.7 (354.8)	2.2 (1037.2)	1.3	0.20–8.55	0.762
Th17A	0.6 (77.1)	1.2 (200.5)	2.0	0.80–4.89	0.129
Th17F	0.6 (69.1)	0.9 (94.8)	1.5	0.75–3.12	0.228
Th22	9.5 (214.6)	13.8 (767.8)	1.5	0.29–7.19	0.63
Myoglobin	178.6 (162.7)	290.7 (229.1)	1.6	0.46–5.81	0.433
S100A8/A9	30.8 (142.8)	207.7 (61.5)	6.8	2.38–19.16	0.001**
Lipocalin A (NGAL)	42.2 (77.0)	68.9 (44.1)	1.6	0.82–3.23	0.15
CRP	65.7 (36.3)	61.2 (16.7)	0.9	0.66–1.32	0.673
MMP-2	38.5 (32.0)	44.9 (6.9)	1.2	0.86–1.57	0.297
Osteopontin	126.5 (75.2)	235.9 (42.9)	1.9	0.96–3.64	0.066
Myeloperoxidase	49.9 (62.9)	63.5 (50.0)	1.3	0.70–2.31	0.407
Serum Amyloid A	374.5 (176.4)	237.9 (53.0)	0.6	0.20–2.03	0.423
IGFBP-4	226.1 (52.3)	310.2 (35.0)	1.4	0.83–2.26	0.199
ICAM1 (CD54)	852.8 (75.0)	1162.0 (90.1)	1.4	0.65–2.86	0.393
VCAM-1 (CD106)	332.5 (66.4)	405.5 (32.9)	1.2	0.67–2.22	0.495
MMP-9	14.0 (223.2)	34.0 (198.4)	2.4	0.59–10.04	0.205
Cystatin C	143.1 (100.1)	189.2 (165.1)	1.3	0.50–3.50	0.555
Angiopoietin-2	651.6 (123.8)	1468.9 (80.4)	2.3	0.84–6.02	0.099
EGF	85.7 (122.8)	205.5 (99.5)	2.4	0.88–6.52	0.083
EPO	20.8 (181.1)	100.1 (139.4)	4.8	1.37–16.91	0.017*
FGF-Basic	18.7 (361.0)	34.2 (221.6)	1.8	0.34–9.85	0.46
G-CSF	19.9 (64.9)	39.4 (49.3)	2.0	1.07–3.64	0.03*
GM-CSF	15.3 (77.1)	26.1 (55.6)	1.7	0.85–3.42	0.126
HGF	65.0 (289.7)	175.1 (260.5)	2.7	0.55–13.22	0.208
M-CSF	23.5 (67.4)	40.1 (582.2)	1.7	0.57–5.10	0.321
PDGF-AA	675.8 (157.6)	1477.4 (144.1)	2.2	0.67–7.15	0.183
PDGF-BB	998.1 (217.0)	2389.8 (116.5)	2.4	0.63–9.12	0.188
SCF	84.5 (59.7)	100.5 (55.0)	1.2	0.66–2.13	0.541
TGF-α	14.4 (50.2)	17.1 (16.3)	1.2	0.75–1.87	0.456
VEGF	6.6 (138.1)	13.2 (345.6)	2.0	0.57–7.12	0.258

Linear regression analysis was used to compare biomarker levels between SJS/TEN survivors and non-survivors groups.

*GM* geometric mean, *GMR* geometric mean ratios, *%CV* percent coefficient of variation.

*Statistically significant with *p* < 0.05, **p < 0.001.

## Discussion

SJS/TEN represents a dermatologic emergency that carries significant morbidity and mortality^13^. Therefore, early and accurate diagnosis, as well as prognostication, are essential for guiding treatment and achieving optimal outcomes^14^. In terms of histopathologic findings, our study did not identify any associations between the degree of epidermal necrosis, the number of dermal mononuclear cell infiltration, and the clinical outcomes. However, an intriguing observation was made regarding the degree of dermal infiltration of CD8 + cells, which displayed a negative correlation with the severity of acute ocular complications. In terms of serum biomarkers, our analysis revealed several associations. Serum IFN-γ levels demonstrated a positive correlation with the number of CD8 + cells. Serum sFasL and SCF levels were found to be elevated in individuals with greater mononuclear cell infiltration in the dermis. Additionally, serum MPO levels exhibited a significant association with the degree of epidermal necrosis. Moreover, S100A8/A9 levels showed a significant correlation with the SCORTEN, and S100A8/A9, G-CSF, and EPO levels correlated with in-hospital mortality rate.

In line with a previous study that encompassed 108 patients diagnosed with SJS/TEN, our investigation did not yield a statistically significant association between the epidermal necrosis and dermal infiltration and hospital mortality^15^. However, contrary to our findings, Quinn et al. conducted a study exclusively focusing on TEN patients. They demonstrated a good concordance between mortality rates and degree of dermal mononuclear cell infiltration, suggesting a potential involvement of inflammatory cells in the severity and prognosis of TEN^16^. The discrepancy observed between the results of prior studies and our own may stem from various factors, including differences in patient classification, mortality rates, and sample sizes. The timing of the skin biopsy is crucial in understanding the observed features.

Our data revealed a negative correlation between the severity of dermal infiltration of CD8 + cells and ocular involvement. Patients with low levels of CD8 + cells exhibited a higher percentage of severe to very severe ocular complications (grade 2–3) compared to those with high levels of CD8 + cells. This correlation aligns with the skin manifestations, as SJS histopathology typically exhibits a higher presence of dermal mononuclear cells, while TEN shows a sparse infiltrate but higher levels of epidermal necrosis. This difference may be explained by the levels of granulysin, which have been reported to be lower in SJS lesions with a higher mononuclear cell count compared to TEN lesions with fewer cells^17,18^). However, it appears biologically challenging to assert that dermal infiltration in the skin is predictive of ocular disease. Further data are required to gain a comprehensive understanding of the relationship between the skin and ocular manifestations.

Numerous studies have explored potential biomarkers for SJS/TEN, including granulysin^19^, Fas ligands (FasL)^20^, perforin B^21,22^, microRNA (miRNA)^23^, annexin A1^24^, S100 calcium-binding protein A2(S100A2)^25^, CCL-27^26,27^, IL-15^28^, galectin-7^29^, RIP3^30^, and high mobility group box 1 protein (HMGB1)^31^. In keeping with the previous reports, our study demonstrated that serum sFasL level was associated with the pathogenesis of SJS/TEN. The differences for other markers, for which we did not find any association with histopathology and clinical outcomes, may stem from differences in the timing of serum/skin samples and the number of recruited cases.

The pathogenesis of SJS/TEN remains incompletely understood, but previous studies have shed light on important molecular and cellular events. Immunopathologic investigations have shown the presence of cytotoxic cells, including CD8 + cytotoxic T lymphocytes (CTLs), natural killer T cells (NKT), and macrophages in early skin lesions of SJS/TEN, suggesting that cell-mediated cytotoxic reactions contribute to extensive keratinocyte apoptosis^32,33^. However, the limited number of infiltrating cytotoxic cells alone cannot account for the widespread full-thickness keratinocyte apoptosis observed. Hence, soluble amplification cytokine mediators may also play a role^34,35^. One hypothesis suggests that costimulatory molecules of antigen-presenting cells (APCs) stimulate CTLs to produce cytokines like IFN-γ and IL-15, ultimately leading to keratinocyte apoptosis^36^. Our findings may support this hypothesis, as we observed CD8 + cells infiltration in SJS/TEN skin lesions and a positive correlation with serum IFN-γ levels.

The Fas–FasL interaction has been widely accepted as one of the pathways involved in the pathogenesis of SJS/TEN^37,38^. FasL is a type-II transmembrane protein belonging to the TNF family. Upon ligand binding, Fas forms the death signaling complex, leading to caspase-8 activation in the cytoplasm and subsequent apoptosis^39^. In a previous study, elevated levels of sFasL were detected in the early stages of SJS/TEN, distinguishing it from healthy controls^20^. Another study suggested that peripheral blood mononuclear cells were the source of increased sFasL production^40^. Our findings demonstrate that SJS/TEN patients with higher levels of mononuclear cell infiltration in the dermis also exhibit elevated serum sFasL levels.

In addition to sFasL, our study revealed elevated serum levels of stem cell factor (SCF) in SJS/TEN patients with increased mononuclear cell infiltration in the dermis. SCF is a dimeric molecule known to act as a growth factor, playing crucial roles in regulating cell viability, proliferation, differentiation, and chemotactic migration^41,42^. While the specific function of SCF in SJS/TEN remains unclear, our findings suggest that SCF may contribute to the pathogenesis of the disease by potentially serving as a cytokine involved in chemotactic migration and cell viability.

Myeloperoxidase (MPO) is a potent oxidative heme enzyme known to induce tissue damage by generating hypochlorous acid and other reactive oxygen species (ROS). It is predominantly stored in azurophilic granules found in polymorphonuclear neutrophils, monocytes, and macrophages^43–45^. A previous study reported elevated MPO levels in blister fluids and serum of patients with TEN, and using dual immunohistochemistry, they identified macrophages as the primary source of MPO in TEN, rather than neutrophils^46^. Interestingly, a recent study has shown that drug-specific CD8 + T cells can induce neutrophil extracellular traps, which initiate the development of SJS/TEN. This finding suggests that neutrophils may have a role in the early phase of the disease^47^. In line with this data, a prior study revealed a significant association between a high neutrophil-to-lymphocyte ratio and increased mortality in SJS/TEN^48,49^. In this present study, we demonstrated a significant correlation between serum MPO levels and the extent of epidermal necrosis in SJS/TEN. These findings suggest that MPO levels could potentially serve as a predictor of epidermal necrosis severity. We did not observe an increase in the number of neutrophils by employing the H&E stain in our skin biopsy specimens. However, this may be due to technical limitations. By employing immunohistochemical stains with MPO and CD66b, the demonstration of neutrophils is improved and more accurate^47^.

S100A8/A9 is a complex of calcium-and zinc-binding proteins released during cell activation and turnover. They are primarily derived from neutrophils and macrophages, and play a crucial role in regulating the inflammatory response by promoting the recruitment of leukocytes and inducing cytokine secretion^50,51^. Prior research demonstrated that S100A8/A9 were expressed in clinically uninvolved skin of SJS/TEN before blister formation^52^. Furthermore, our study revealed a significant inverse correlation between serum S100A8/A9 levels and the number of disease survivors. Intriguingly, in this study, the degree of epidermal necrosis did not show a significant correlation with the in-hospital mortality rate. Therefore, in this context, serum S100A8/A9 may serve as a potential prognostic indicator and aid in the triage and placement of patients in the intensive care unit.

In the non-survivor group, significantly higher levels of G-CSF and EPO were observed. The potential role of G-CSF and EPO in the pathogenesis of SJS/TEN has not been previously reported. We speculate that the increase in G-CSF and EPO might be attributed to the heightened inflammatory responses of myeloid and erythroid cells, possibly reflecting an uncontrolled cytokine storm in this patient group. Nevertheless, two cases of TEN with concurrent neutropenia showed promising clinical outcomes following the administration of G-CSF. G-CSF was found to promote faster re-epithelialization, facilitating the accelerated regeneration of damaged tissues^53^. This illustrates that the role of both cytokines in the disease is complex, and the precise impact of these biomarkers on the mortality rate requires further investigation to be fully understood.

There are several limitations in this study. Firstly, the rarity of SJS/TEN has hindered the ability to conduct controlled clinical trials. Instead, we used a retrospective design that can only establish associations, not causal relationships. Secondly, the presence of confounding factors, such as variations in comorbidities and treatment choices, may influence the variability in serum and biopsy results and potentially impact mortality rates. Thirdly, it is important to note that this study was conducted at a single tertiary care center with a limited sample size. Additionally, it is essential to acknowledge that CD4 can be expressed by macrophages in human skin, so a single CD4 stain could represent both T cells and/or macrophages. Similarly, activated CD8 cells can express CD56, so CD56 is not a perfect marker for NK cells. These considerations are crucial when interpreting and generalizing the results related to these markers in the study. Moreover, the biomarkers examined in this study are serum proteins. However, whether these proteins are elevated in skin/blister fluid was not tested. Finally, the mean ± SD time from disease onset to skin biopsy was 4.82 ± 2.40 days, and 4.35 ± 2.46 days for serum collection, as some of the patients were referred from other hospitals, and there was a delay in seeking medical care in some patients. Taking samples earlier in the disease would, therefore, be more useful to identify biomarkers that can predict the disease progression.

The mortality rate seemed high in the current study, with six non-survivors (26.1%). This is because those who died were older (mean age 61.17 ± 11.55 years) and had significant underlying diseases. Four out of six patients had malignancy, while the other two suffered from cardiomyopathy and connective tissue disease. The most common cause of death was infection/sepsis, highlighting the importance of supportive care and infection prevention measures in these patients.

## Conclusions

Specific serum biomarkers, such as IFN-γ, sFasL, MPO, and SCF, exhibited significant associations with the histologic characteristics of SJS/TEN. Additionally, the levels of S100A8/A9 showed a positive correlation with the severity score assessed by SCORTEN and the in-hospital mortality rate. These findings provide valuable insights into the intricate pathogenesis of the disease, potentially leading to the development of targeted therapies.

## Materials and methods

### Study design and ethical considerations

This study was a retrospective study conducted between March 2015 and July 2021 at King Chulalongkorn Memorial Hospital, Bangkok, Thailand. The study protocol was approved by the Institutional Review Board, Faculty of Medicine, Chulalongkorn University (IRB No. 071/60) and was conducted following the principles of the Declaration of Helsinki and the strengthening the reporting of observational studies in epidemiology (STROBE) reporting guideline (Supplementary). All data were obtained by reviewing the medical records and electronic database of the Thailand Severe Cutaneous Adverse Reactions (ThaiSCAR) cohort registered at ClinicalTrials.gov (NCT02574988). The participants provided informed consent and the data was deidentified.

### Study population and data collection

Patients with SJS/TEN aged more than 18 years old were eligible. The diagnosis and classification of SJS/TEN were based on established consensus criteria with supportive histological evidence^54^. The suspected culprit drugs were identified according to the algorithm of drug causality for epidermal necrolysis (ALDEN)^55^. According to the ThaiSCAR protocol, patients were admitted to the hospital during the acute phase within 8–12 days of symptom onset. Detailed medical information (relevant demographics, comorbidities, causative drugs, percentage of BSA detachment, SCORTEN, in-hospital mortality), serum samples, and skin biopsy specimens were collected on admission.

### Serum biomarkers analysis

The serum samples of patients (30 ml) were collected within a maximum of 3 days following admission. Undiluted samples were stored at − 80 °C until biomarker measurement. After thawing, the samples were analyzed using a bead-based multiplex immunoassay. Panels of 42 biomarkers, including the Human Th17 Cytokine Panel 7-plex (IL-6, IL-10, IFN-γ, TNF-α, Th17A, Th17F, and Th22), the Human Growth Factor Panel 13-plex (Angiopoietin-2 (Ang-2), EGF, EPO, FGF-basic, G-CSF, GM-CSF, HGF, M-CSF, PDGF-AA, PDGF-BB, SCF, TGF-α and VEGF), human vascular inflammation panel 13-plex (Myoglobin, Calprotectin (S100A8/A9), Lipocalin A (NGAL), C-Reactive Protein (CRP), MMP-2, Osteopontin (OPN), Myeloperoxidase (MPO), serum amyloid A (SAA), IGFBP-4, ICAM-1 (CD54), VCAM-1 (CD106), MMP-9, and Cystatin C), and the custom Human panel 9-plex (TGF-b1, IL-18, IP-10, MCP-1, sFASL, IL-15, Rantes, IL-23, and Granulysin) were used to identify biomarkers for SJS/TEN.

To simultaneously quantify soluble analytes in plasma samples, 25 μl of assay buffer was added to each well. The standard or sample was mixed with 25 μl of diluted standard or plasma. Then, 25 μl of mixed beads and 25 μl of detection antibodies were added to each well and incubated for 2 h at room temperature on an orbital plate shaker. After incubation, 25 μl of the streptavidin-PE solution was added and then incubated for 30 min at room temperature on an orbital plate shaker. After the liquid was decanted and washed a second time with wash buffer, 150 μl of wash buffer was added to each well and shaken for 2–3 min prior to flow cytometric analysis (BD FACSCaliburTM, Becton Dickinson, USA).

### Histologic examination

Tissue samples were obtained from the lesional skin (dusky red area without epidermal detachment) of individuals using a 4-mm punch biopsy. The procedures were performed by dermatologists who were prior trained to choose a proper and similar site of biopsy, according to the ThaiSCAR protocol. In the first part, tissue sections were stained with hematoxylin–eosin (H–E) and critically reviewed by three dermatopathologists (J. W., P. C., and C. S.). The degree of epidermal necrosis and dermal mononuclear cell infiltration were graded as mild, moderate, and extensive by the majority consensus evaluation (at least 2 out of 3 dermatopathologists agreed on the severity). The full-thickness epidermal necrosis was considered extensive, the partial thickness was moderate, and sparse scattering with focal necrotic keratinocytes was considered mild. Examples of histology grading are shown in Fig. S1. For cases with total disagreement, the slides were reviewed to achieve consensus. In the second part, immunohistochemical analyses were performed using the Ventana Benchmark ULTRA system to determine the CD4, CD8, CD56, CD68, CD117, and granzyme B-expressing cells in the tissue. The Membranous v.9 algorithm (Aperio Technologies) was chosen to quantify the positivity of the staining; positive staining cells (cell/mm^2^) = (percentage of positive cells x total cell analyses)/total area of the entire dermis.

### Statistical analysis

Descriptive, categorical variables were reported as frequency and percentage, while continuous variables were reported as mean and standard deviation (SD). The association between histological features and clinical severity measures, including SCORTEN, percentage of BSA involvement, ocular severity score, in-hospital mortality rate, underlying disease, and causative drugs, was evaluated using the Chi-square test. Additionally, similar analyses were conducted to examine the correlation between the intensity of immunostaining and these clinical severity parameters. Serum biomarker levels were log-transformed and described as geometric means (GM) with the percent coefficient of variation (%CV). Linear regression analysis was performed to assess the differences in logarithmic mean values of serum biomarker levels between groups, and the results were reported as geometric mean ratios (GMR). The Spearman correlation was used to evaluate the correlation between serum biomarker levels and the SCORTEN severity score. All analyses were performed using STATA version 15.1 (StataCorp LLC., Texas, USA). The statistical significance threshold was set at a *P*-value of 0.05. Scatter plot graphs were created by GraphPad Software.

### Supplementary Information

Supplementary Figure S1.

## Data Availability

All data is not available in the manuscript and supplementary material but can be made available from the corresponding author on reasonable request.
