# Supplementary material for: Correlations between histopathologic findings, serum biomarker levels, and clinical outcomes in Stevens–Johnson syndrome/toxic epidermal necrolysis (SJS/TEN)
Source: Sci Rep. 2023 Aug 21;13:13620. doi: 10.1038/s41598-023-40812-3 (PMC10442337; doi:10.1038/s41598-023-40812-3)
Supplement: Supplementary file 1 — Supplementary Figure S1. [file 41598_2023_40812_MOESM1_ESM.docx]

**Level of keratinocyte necrosis**


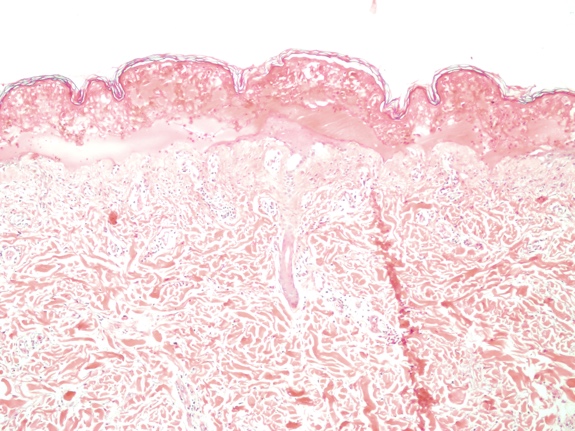
 Full thickness necrosis


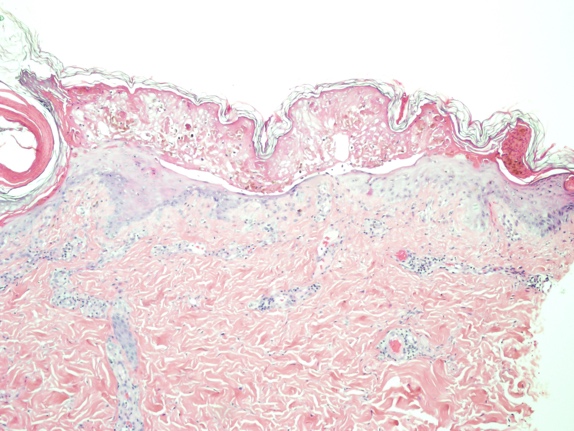
 Partial thickness


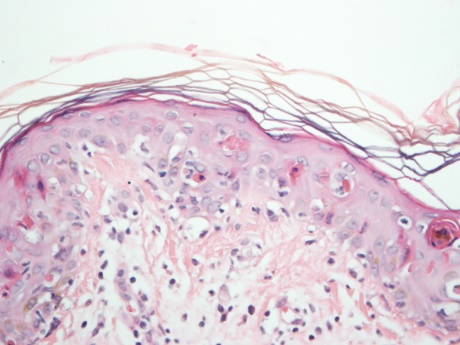
 Sparse (scatter)


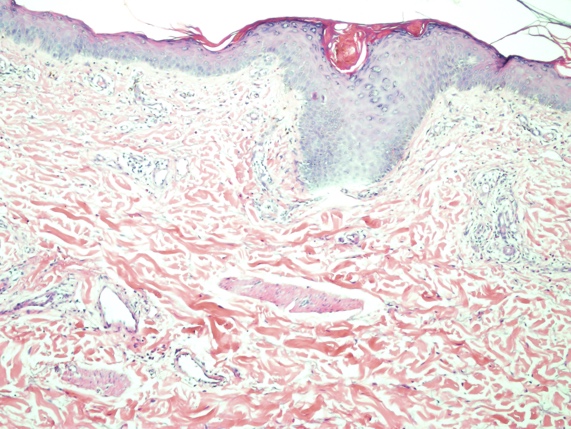
 Sparse (focal)

**Degree of mononuclear cell infiltration**


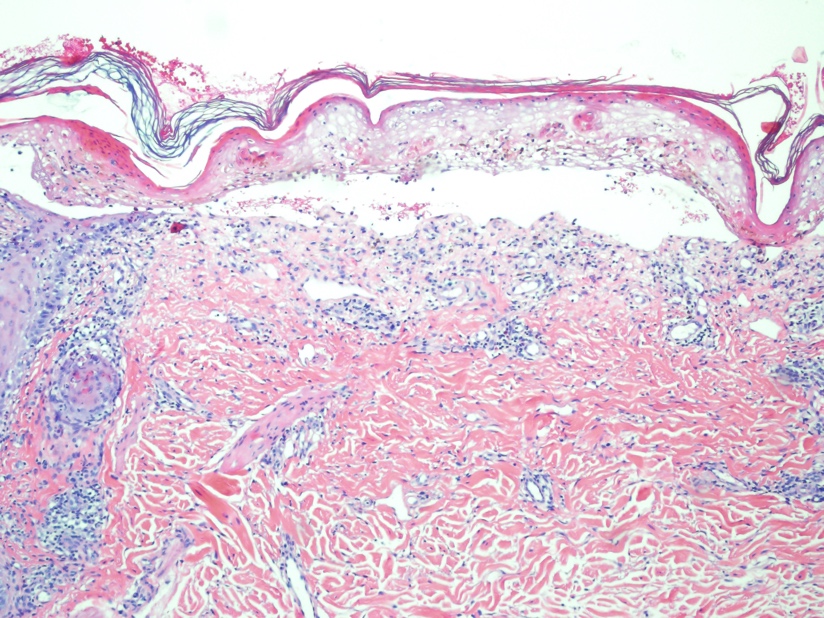
 Extensive cell infiltration
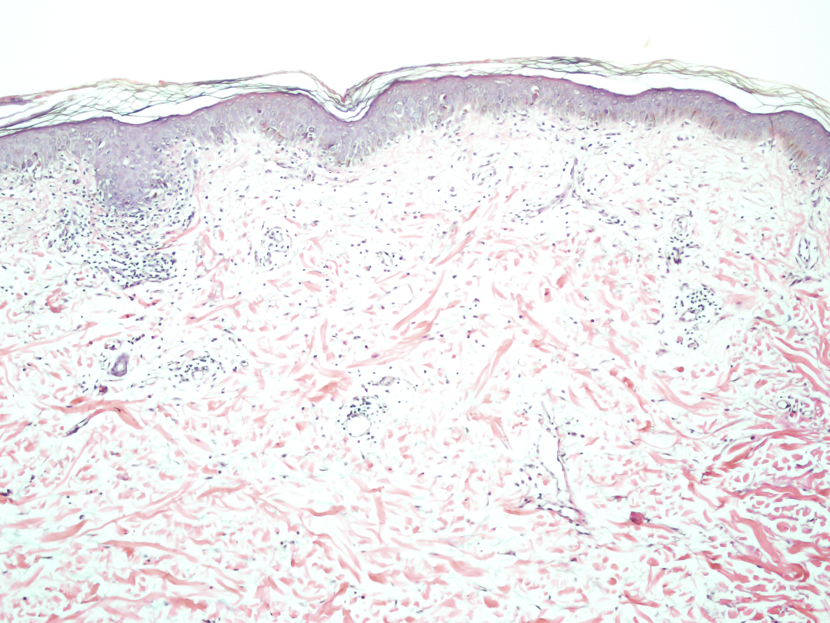
 Moderate cell infiltration
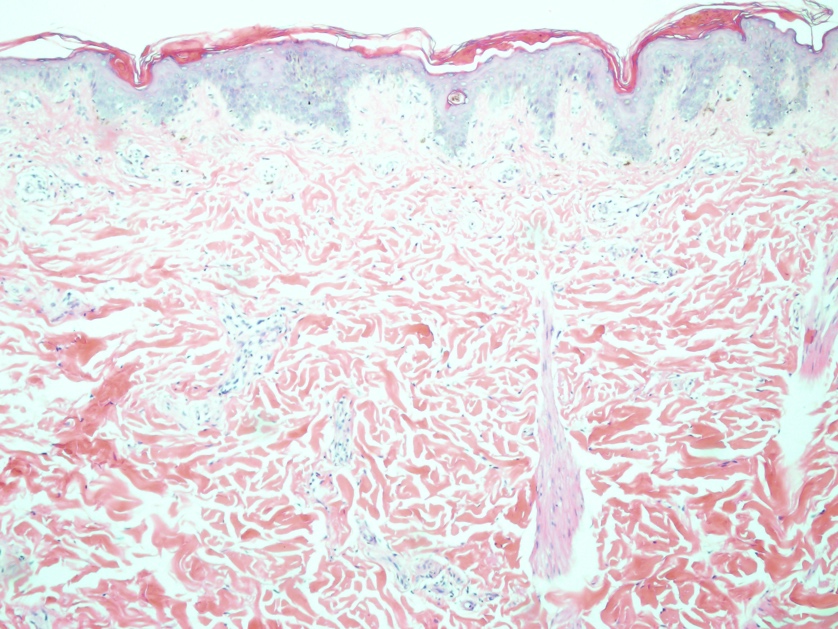
 Mild, sparse

**Fig . S1.** Examples of histology grading. The full-thickness epidermal necrosis was considered extensive, the partial thickness was moderate, and sparse scattering with focal necrotic keratinocytes was considered mild. The degrees of dermal mononuclear cells infiltration were graded as mild, moderate, and extensive according to the number of cell infiltration as shown. (Haemotoxylin and Eosin stain, magnification 200X)
